# Effectiveness of Needle Tenotomy for Correction of Equinus in Clubfoot

**DOI:** 10.7759/cureus.32080

**Published:** 2022-11-30

**Authors:** Ripudaman Sharma, Arvind Kumar, Siddhartha Sinha, Javed Jameel, Rizwan Khan, Sandeep Kumar

**Affiliations:** 1 Orthopaedics, GS Medical College and Hospital, Pilkhuwa, IND; 2 Orthopaedics, Maulana Azad Medical College, New Delhi, IND; 3 Orthopaedics, Hamdard Institute of Medical Sciences and Research, New Delhi, IND; 4 Orthopaedics, KG Medident Medical and Dental Care Center, Ghaziabad, IND

**Keywords:** tenotomy, tendoachilles, equinus, deformity, clubfoot, achilles tendon

## Abstract

Introduction: During the COVID-19 surge, due to a lack of operating room availability, we performed Achilles tendon tenotomy in clubfoot patients using a 16/18 gauge needle to avoid delay in their management. The procedures were performed on an outpatient basis. The current retrospective study aims to investigate the effectiveness of needle tenotomy for the correction of equinus in clubfoot at a minimum of one year of follow-up.

Methods: Clinical records of all clubfoot patients that underwent needle tenotomy of Achilles tendon from March 2020 onwards with at least one year of follow-up were reviewed. We recorded Pirani scores and the equinus deformity at the initial presentation, after Achilles tendon tenotomy, and at the final follow-up. We also recorded any procedure-related complications following the Achilles tendon tenotomy. We compared dorsiflexion after final cast removal and after one year of follow-up.

Results: A total of 26 clubfeet in 14 patients underwent needle tenotomy of the Achilles tendon and completed one year of follow-up. Ankle dorsiflexion was achievable in all patients and the mean dorsiflexion of 27.4 degrees. The average Pirani score after tenotomy at final cast removal was 0.16, while the mean dorsiflexion at final cast removal was 24.2 degrees (p = .00084). No tenotomy procedure-related complications were noted.

Conclusion: Percutaneous needle tenotomy of the Achilles tendon is a simple, safe, and effective technique for equinus correction in clubfoot. Considering the less invasive nature of the procedure, it can be done as a short procedure on an outpatient basis and has a limited risk of hemorrhage and other wound-related complications.

## Introduction

Ponseti casting and manipulation is currently the standard treatment for congenital talipes equinovarus (CTEV) [1]. This method has been accepted worldwide as the most effective form of management, both in fresh cases and late presenting ones [1]. The cavus is corrected first, followed by simultaneous correction of adduction and varus deformities. The equinus component, which is last to be corrected, often requires tenotomy of the Achilles tendon to reach maximum dorsiflexion. Forced dorsiflexion without Achilles tendon tenotomy can result in a midfoot break [2]. The Achilles tenotomy is the only invasive procedure in the standard management of clubfoot. The tenotomy is usually performed as an outpatient percutaneous procedure. However, several authors have recommended a mini-open procedure in the operating room (OR) to allow better visualization of the tendon and avoid damage to the local structures [3-5]. During the surge of the COVID-19 pandemic, when prolonged lockdowns were ordered, and routine surgeries were postponed, our department was also affected both by manpower and resources. During that period, due to lack of OR availability, we performed Achilles tendon tenotomy in clubfoot patients using a 16/18 gauge needle to avoid delay in their deformity management. The procedures were performed on an outpatient basis. This study aims to investigate the effectiveness of needle tenotomy of the Achilles tendon for correction of equinus in clubfoot and its maintenance at a minimum of one year of follow-up.

## Materials and methods

After approval from the institutional review board of Hamdard Institute of Medical Sciences and Research, New Delhi (approval no.: not applicable (exempted)), we retrospectively reviewed the clinical records of all clubfoot patients that underwent needle tenotomy of the Achilles tendon from March 2020 onwards with at least one year of follow-up. All cases were reviewed irrespective of early or delayed presentation, age, and etiology of clubfoot. We recorded the baseline demographic characteristics of all patients, such as age, gender, laterality, etiology, and Pirani scores and their equinus component at the initial presentation. Additionally, the equinus component values after Achilles tendon tenotomy and at last follow-up were measured. We also recorded any procedure-related complications following the Achilles tendon tenotomy. The inability to correct the equinus deformity with needle tenotomy was recorded as a failure of the technique. The continuous variables were expressed as mean (range), and the discrete variables were expressed as proportions. We used Wilcoxon signed-rank test to compare dorsiflexion correction after final cast removal and after one year of follow-up.

Surgical technique

**Figure 1:**
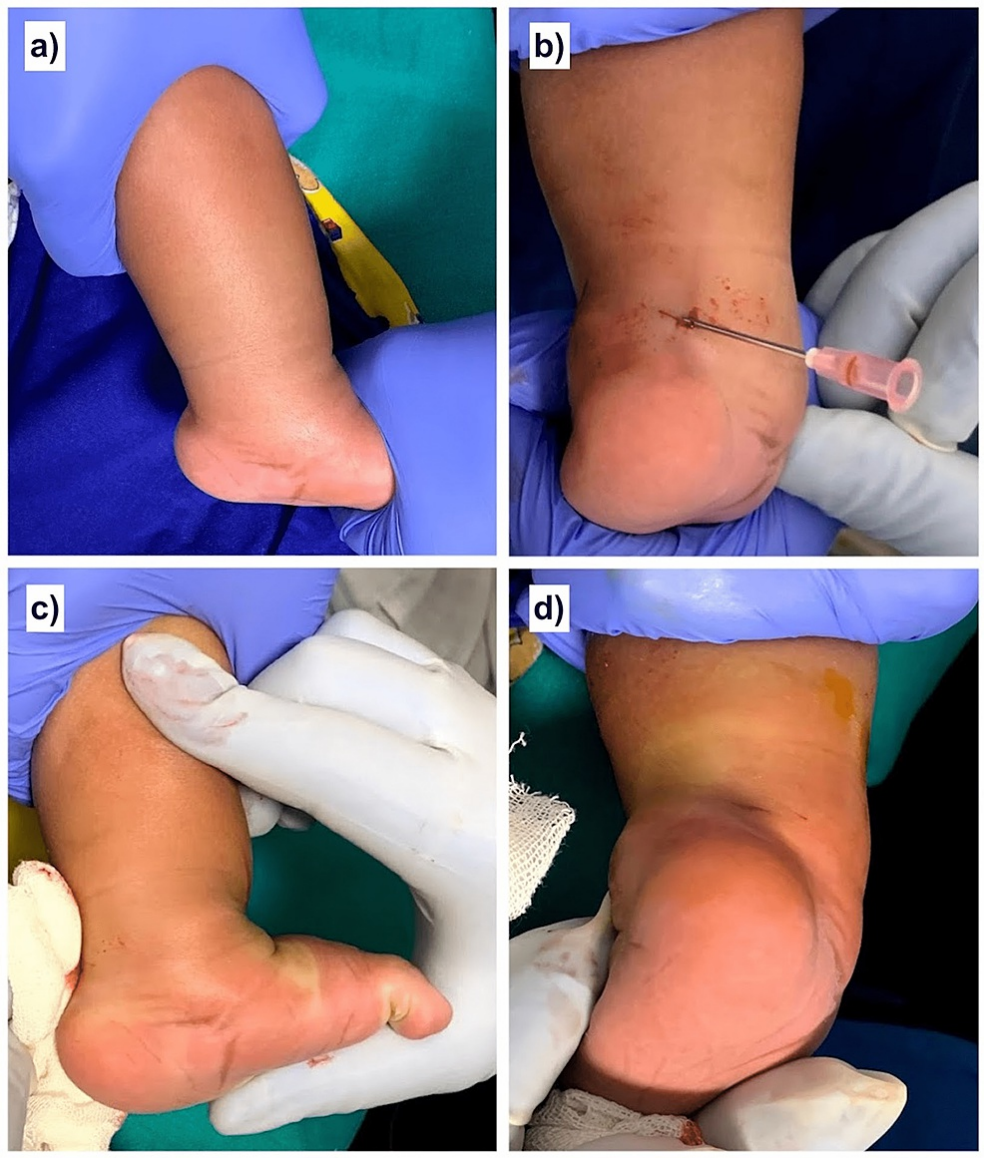
Stepwise demonstration of needle tenotomy procedure of Achilles tendon tenotomy a) Foot is gently dorsiflexed to make the Achilles tendon taut and its medial border easily palpable b) An 18 gauge needle is inserted along the medial border of the tendon approximately one finger width above its insertion, and its tip is toggled anteroposterior while gradually moving from medial to lateral until a snap of complete tenotomy is felt c) The needle is removed, the foot is kept in maximum dorsiflexion, and the tenotomy site is compressed to prevent any hematoma formation d) Final dressing and casting procedure is done when there is no ooze from the needle entry site

Achilles tendon tenotomy (Figure 1) was planned after the midfoot Pirani score was corrected to zero or could not be corrected further for non-zero values following serial manipulation and casting according to the Ponseti method. Under all aseptic precautions and oral analgesic support, the knee was flexed to 90 degrees, and the foot was applied a dorsiflexion force to keep the Achilles tendon taut. Approximately 1 ml to 2 ml of local anesthetic solution was infiltrated on the palpable medial border of the Achilles tendon at the desired tenotomy site, which was approximately one finger width proximal to the Achilles tendon insertion. A 16/18 gauge needle was inserted through the infiltrated site and was toggled from anterior to posterior direction while feeling the tendon surface through its sharp tip while gradually moving from medial to lateral until a snap of complete tenotomy was appreciated with a sudden release of ankle dorsiflexion. The contact between the needle tip and the tendon surface was used as a guide to localize the anteroposterior and mediolateral extent of the tendon. The anteroposterior toggling movement helped in cutting taut tendon fibers. The foot was kept maximally dorsiflexed, and the tenotomy site was compressed for a few minutes to prevent any hematoma formation and till there is no blood ooze from the needle entry site. A palpable dip in the Achilles tendon contour was a sign of tendon discontinuity, while the persistence of palpable tendon and difficulty in dorsiflexion beyond neural were perceived as incomplete tenotomy. The same tenotomy steps were repeated in such cases until a snap or pop was appreciated with the release of dorsiflexion beyond the neutral position. A small sterile gauze dressing was done, and a corrective above-knee cast was applied in maximum dorsiflexion for three weeks. Thereafter, the patients were kept on clubfoot bracing protocol. The foot correction was maintained using a Steenbeek brace or a customized foot abduction brace. The brace was advised for full-time wearing except during baths and foot cleaning for the first three months after tenotomy cast removal. Thereafter, a night and nap bracing frequency was advised totaling up to 14 to 16 hours per day. This protocol was continued for four years.

## Results

A total of 26 idiopathic congenital clubfeet in 14 patients underwent needle tenotomy of the Achilles tendon and had completed at least one year of follow-up. The mean follow-up period was 13.9 (12 to 16) months. There were 12 male and two female patients. The mean age at the initial presentation was 6.8 months. Twelve patients had both feet involvement, and two had unilateral. The mean initial Pirani scores were 5.28 (4.5-6), while the mean equinus deformity was 28.4 (18-42) degrees. All patients had an equinus component of one at the initial presentation. Mean 5.6 (5-8) casts were applied before the tenotomy. The mean Pirani score before Achilles tenotomy was 1.73 (1.5-2). The equinus component was "1" in all cases. The mean Pirani score after tenotomy at final cast removal was 0.16 (0-0.5), while the equinus component was zero in all patients. The mean dorsiflexion at final cast removal was 24.2 (18-31) degrees. After one year of follow-up, the mean Pirani score was 0.41 (0-1). The mean follow-up duration was 16.5 months. Ankle dorsiflexion was achievable in all patients and the mean dorsiflexion of 28.34 (22-36) degrees. The ankle dorsiflexion was found to be significantly higher at the final follow-up compared to the dorsiflexion correction at final cast removal (p = .00084). There were no failures, and no tenotomy procedure-related complications were noted. The case details are shown in Table 1.

**Table 1:** Details of cases included in the current series

Case number	Age in months	Affected side (right/left)	Initial Pirani score (0-6)	Initial Equinus component (0/0.5/1)	Pirani score before tenotomy (0-6)	Pirani Score at final cast removal (0-6)	Equinus component at final cast removal (0/0.5/1)	Pirani score at the last follow-up (0-6)	Initial equinus deformity (in degrees)	Dorsiflexion at final cast removal (in degrees)	Dorsiflexion at last follow-up ( in degrees)	Number of casts required before tenotomy	Follow-up duration (in months
Case 1	3	Right	5.5	1	1.5	0.5	0	1	30	21	23	6	16
		Left	5	1	1.5	0.5	0	0.5	27	26	28	6	
Case 2	5	Right	6	1	1.5	0.5	0	0.5	26	22	30	6	15
		Left	5.5	1	1.5	0	0	0.5	32	28	27	6	
Case 3	9	Right	5.5	1	2	0	0	0.5	30	31	32	6	13
		Left	5.5	1	1.5	0	0	0	27	27	22	7	
Case 4	11	Right	5.5	1	2	0	0	0	32	22	27	6	12
		Left	6	1	2	0.5	0	0.5	32	24	34	8	
Case 5	4	Right	6	1	1.5	0	0	0.5	42	25	36	5	13
		Left	5.5	1	2	0	0	0.5	41	27	28	5	
Case 6	7	Right	5.5	1	2	0	0	0.5	36	25	32	5	13
		Left	4.5	1	1.5	0.5	0	0.5	28	22	27	5	
Case 7	9	Right	4.5	1	2	0.5	0	1	22	19	36	5	14
		Left	5	1	1.5	0.5	0	0.5	20	28	24	5	
Case 8	4	Right	5	1	2	0	0	0	18	20	26	5	14
		Left	4.5	1	1.5	0	0	0	21	22	34	5	
Case 9	5	Right	4.5	1	1.5	0	0	0	20	23	36	5	15
		Left	4.5	1	1.5	0	0	0.5	32	22	24	5	
Case 10	6	Right	4.5	1	2	0	0	0.5	32	27	26	5	12
		Left	4.5	1	1.5	0	0	0.5	27	29	28	5	
Case 11	10	Right	5.5	1	2	0	0	0	22	31	32	5	15
		Left	5.5	1	1.5	0	0	0	26	21	26	6	
Case 12	5	Right	6	1	2	0.5	0	1	29	20	28	6	15
		Left	6	1	2	0	0	0.5	31	18	25	6	
Case 13	7	Right	5.5	1	1.5	0	0	0	36	26	24	6	16
Case 14	11	Right	6	1	2	0	0	0.5	21	23	22	7	12

## Discussion

The current series of findings suggests percutaneous needle tenotomy is a safe and effective alternative to conventional tenotomy techniques for Achilles tendon release in congenital clubfoot. The procedure, performed through a small 16/18 needle hole, circumvents the need for stab incision and hemostasis in case of injury to nearby vascular branches. The intact skin itself potentially acts as a compression bridge over the tenotomy site. 

Originally, a scalpel blade was used for Achilles tendon tenotomy in the Ponseti method for clubfoot management. Due to its larger area and length of the sharp surface, the blade carries the risk of injury to local neurovascular structures [6]. Burghardt et al. reported the risk of pseudoaneurysm with the use of blade tenotomy [7]. The complications were likely to be due to the percutaneous nature of the procedure, which limits the accurate visualization of the tendon and surrounding procedure. The sharp edges of the blade could not be confined to a point location and thus had a risk of injuring nearby vessels. For better visualization, mini-open techniques have been suggested in which a larger incision is made to visualize and secure the tendon [3-5]. This helps in the precise tenotomy of the Achilles tendon due to adequate localization of its margins. The vascular anatomy posterior to the ankle can also vary, resulting in additional risk with a blind procedure. There have been reports in which peroneal vessels have been reported to be a major source of foot and ankle circulation [7,8]. An injury to these vessels can thus comprise foot and ankle circulation in such circumstances. 

Needle tenotomy offers a safer option for tenotomy, considering the sharp cutting zone is very small and can remain localized to the tendon, unlike the blade, where the sharp zone is longer and can encounter surrounding structures. The technique has been previously tested by some authors for the tenotomy of the Achilles tendon in clubfoot management. Maranho et al. reported it to be a safe tenotomy option, with only two patients out of 57 having complications of minor bleeding [9]. Evans et al. also observed only minor bleeding complications in 11 out of 425 that were controlled with pressure over the wound [10]. Rahman et al. also observed similar complications in nine out of 70 club feet [11]. Interestingly, the ankle dorsiflexion improved significantly after a one-year follow-up. The current literature evidence suggests that ankle dorsiflexion gradually decreases with time in clubfoot [12]. We feel that a shorter follow-up of one year and a limited number of patients could have been the reason for such an observation. In addition, the difference in dorsiflexion was ~4 degrees which might not be clinically relevant, even if statistically significant. 

Although equinus correction was achieved in all patients in our series, we feel that there might be the risk of incomplete tendon release when performed by surgeons non-familiar with this technique. To circumvent this issue, surgeons can practice on cadaveric specimens for needle-based tendon release. Maranho et al. used ultrasonography to visualize the completeness of needle tenotomy for clubfoot [9]. However, such facilities are not available in all setups and may incur additional costs. 

The series has major limitations of small sample size and retrospective nature. Moreover, there would have been several other factors influencing the outcomes related to equinus correction, like the stiffness of the deformity, compliance with the bracing protocol, and duration of follow-up. Those were not analyzed in this series. In addition, the series presents the experience of one center and the reproducibility may vary from surgeon to surgeon. However, the series strengthens the generalized use of needle tenotomy as a safe option in clubfoot management. With wider acceptability, the needle tenotomy can be potentially used as a standard treatment of equinus contracture in clubfoot.

## Conclusions

In the current series, we share our experience of needle tenotomy for Achilles tendon release in clubfoot deformity management. Percutaneous needle tenotomy of the Achilles tendon is a simple, safe, and effective technique for equinus correction in clubfoot. Considering the less invasive nature of the procedure, it can be done as a short procedure on an outpatient basis and has a limited risk of hemorrhage and other wound-related complications. Furthermore, the equinus correction is maintained at a one-year follow-up. The needle used in the tenotomy technique is an easily available resource and can potentially be used to train health personnel in remote areas to counter the problem of neglected clubfoot in developing countries. However, more evidence in the form of prospective comparative studies will be needed to support the routine use of needle tenotomy as a standard procedure in clubfoot management.
